# HPIP and RUFY3 are noncanonical guanine nucleotide exchange factors of Rab5 to regulate endocytosis-coupled focal adhesion turnover

**DOI:** 10.1016/j.jbc.2023.105311

**Published:** 2023-10-04

**Authors:** Saratchandra Singh Khumukcham, Vasudevarao Penugurti, Suresh Bugide, Anju Dwivedi, Anita Kumari, P.S. Kesavan, Sruchytha Kalali, Yasaswi Gayatri Mishra, Vakkalagadda A. Ramesh, Hampapathalu A. Nagarajaram, Aprotim Mazumder, Bramanandam Manavathi

**Affiliations:** 1 Department of Biochemistry, School of Life Sciences, University of Hyderabad, Hyderabad, Telangana, India; 2 Department of Biological Sciences, Tata Institute of Fundamental Research (TIFR), Hyderabad, Telangana, India; 3 Laboratory of Computational Biology, Centre for DNA Finger Printing and Diagnostics (CDFD), Hyderabad, Telangana, India; 4 Laboratory of Computational Biology, Manipal Academy of Higher Education, Manipal, Karnataka, India; 5 Department of Systems and Computational Biology, University of Hyderabad, Hyderabad, Telangana, India

**Keywords:** HPIP/PBXIP1, Rab5, RUFY3, focal adhesion kinase, cell migration, endocytosis

## Abstract

While the role of endocytosis in focal adhesion turnover-coupled cell migration has been established in addition to its conventional role in cellular functions, the molecular regulators and precise molecular mechanisms that underlie this process remain largely unknown. In this study, we report that proto-oncoprotein hematopoietic PBX–interacting protein (HPIP) localizes to focal adhesions as well as endosomal compartments along with RUN FYVE domain–containing protein 3 (RUFY3) and Rab5, an early endosomal protein. HPIP contains two coiled-coil domains (CC1 and CC2) that are necessary for its association with Rab5 and RUFY3 as CC domain double mutant, that is, mtHPIPΔCC1-2 failed to support it. Furthermore, we show that HPIP and RUFY3 activate Rab5 by serving as noncanonical guanine nucleotide exchange factors of Rab5. In support of this, either deletion of coiled-coil domains or silencing of HPIP or RUFY3 impairs Rab5 activation and Rab5-dependent cell migration. Mechanistic studies further revealed that loss of HPIP or RUFY3 expression severely impairs Rab5-mediated focal adhesion disassembly, FAK activation, fibronectin-associated-β1 integrin trafficking, and thus cell migration. Together, this study underscores the importance of HPIP and RUFY3 as noncanonical guanine nucleotide exchange factors of Rab5 and in integrin trafficking and focal adhesion turnover, which implicates in cell migration.

Endocytosis is an essential cellular process that regulates numerous signaling pathways by controlling cell surface receptors. It is increasingly evident that endocytosis modulates cell adhesion signaling *via* integrins, receptors for extracellular matrix proteins, to control cell migration (1, 2). During cell migration, integrins are internalized from the cell surface *via* endocytosis and recycled back to the plasma membrane to redistribute the pools of integrin from one part of the cell to another (3, 4, 5). It has been known that the internalization of integrins *via* clathrin-mediated endocytosis regulates the turnover of focal adhesions (FAs) (6, 7). In this context, focal adhesion kinase (FAK) has been recognized as a key player in FA signaling/turnover and clathrin-mediated endocytosis as FAK-null cells display impaired FA turnover and decreased cell migration (6, 8, 9). While FAK activation is a prerequisite for endosome-mediated FA turnover and cell migration, the molecular regulators of FAK activation and coordinators of this process remain elusive.

Endosomal proteins, such as Rab5 and early endosome antigen 1, are essential players in the generation and propagation of early endosomes during endocytosis (10, 11). While endocytosis is associated with cell migration, in particular, the role of Rab5 in cell adhesion and migration has been pertinent. Several studies revealed that Rab5 regulates cell migration *via* cytoskeletal remodeling that results in Rac1 activation (12, 13, 14), β1 integrin internalization and recycling (13), or microtubule (MT)-dependent adhesion disassembly (15). While the current concepts envisage the crucial role of Rab5 in endocytosis-mediated adhesion signaling *via* integrins, the molecular regulators of Rab5 activity and the detailed mechanisms that underlie this process are not fully understood.

Hematopoietic PBX–interacting protein (HPIP) is an oncoprotein with an adaptor function that can integrate various signaling proteins in the regulation of cell migration (16, 17, 18, 19, 20, 21, 22, 23, 24). Its overexpression has been reported in various cancers (25). Not only that its overexpression is implicated in the development of osteoarthritis (26). Recent studies revealed a novel signaling axis involving HPIP and Talin in FA turnover and cell migration (27). While there is increasing evidence regarding its role in cell migration, its precise molecular mechanism in FA signaling and cell migration is elusive (27).

In this report, we have characterized HPIP and RUN FYVE domain–containing protein 3 (RUFY3) as noncanonical guanine nucleotide exchange factors (GEFs) of Rab5 that regulate endocytosis-coupled FA turnover and cell migration. Through coiled-coil (CC) domains HPIP connects with Rab5 and RUFY3. This trimolecular molecular association triggers FAK activation, FA disassembly, and fibronectin (FN) associated-β1 integrin trafficking, and thus cell migration.

## Results

### Identification of RUFY3 as HPIP-interacting protein

**Figure 1:**
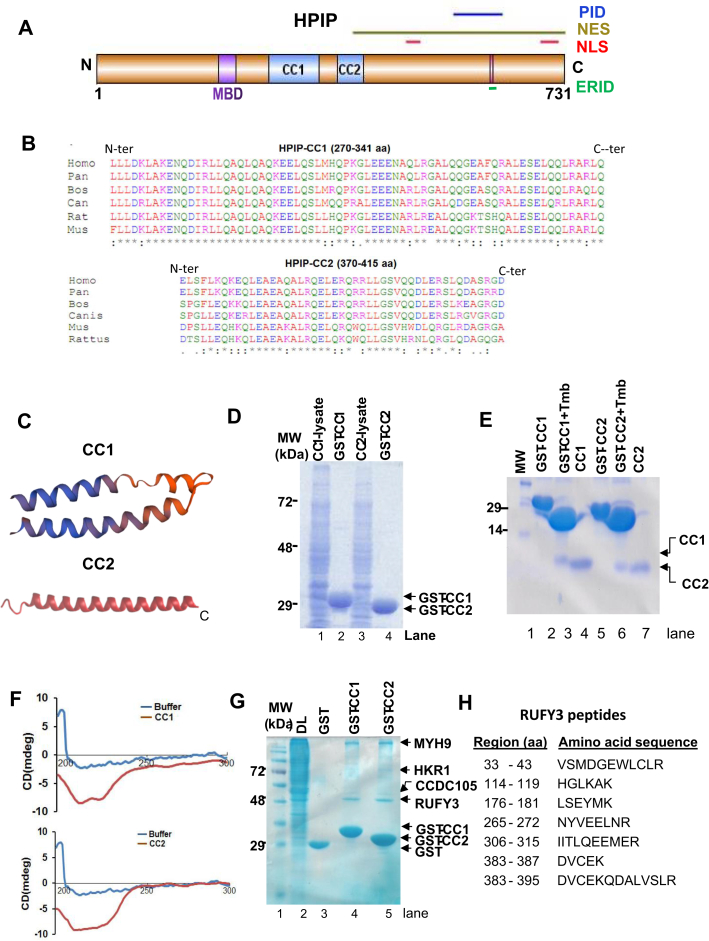
**Identification of RUFY3 as a HPIP-interacting protein.***A*, physical map of HPIP displaying various functional domains. *B*, multiple sequence alignment by Clustal W tool showing the conservation of CC domains of HPIP in various species. *C*, protein structure of CC domains predicted by I-TASSER software. *D*, SDS-PAGE analysis of recombinant GST-CC1 and GST-CC2 domains of HPIP, which are expressed and purified in *Escherichia coli* (DE3). *E*, affinity purified GST-tagged CC1 and CC2 were given thrombin (Tmb) treatment to remove GST tag and after thrombin removal, CC1 and CC2 were separated on SDS-PAGE. *F*, purified CC1 and CC2 domains in PBS were subjected to CD spectra analysis (*upper panel*). *G*, GST pull-down assay was performed to identify HPIP (bait)-interacting proteins using MDA-MB231 protein extract (prey). Unique protein bands were excised from the gel and subjected to MALDI-TOF analysis and identified proteins are MYH9, HKR1, CCDC105, and RUFY3. *H*, RUFY3 peptides identified in MALDI. CC1 and CC2, coiled-coil domains 1 and 2; ERID, ERα-interacting domain; HPIP, hematopoietic PBX–interacting protein; NES, nuclear export signal; NLS, nuclear localization signal; PID, PBX1-interacting domain; RUFY3, RUN FYVE domain–containing protein 3.

Our earlier studies defined the role of HPIP in cell migration (27). In the present study, we sought to further understand the mechanism underlying HPIP-mediated cell migration. HPIP protein contains few distinct functional domains, two carboxy-terminal nuclear localization signals (485–505 and 695–720 aa), nuclear export signal (402–731 aa), PBX1-interacting domain (560–633 aa), MT-binding domain (190–218 aa), and estrogen receptor alpha–interacting domain (615–619 aa) (17, 28) (Fig. 1*A*). In addition to these known domains, *in silico* analysis (COILS server of Expasy) revealed two highly conserved CC domains in HPIP, which are CC1, 270 to 341 aa and CC2, 370 to 415 aa, and they are well conserved across the species (Figs. 1*B* and S1*A*). Further protein modeling studies showed that CC1 configures three unequal helices connected by spacers, whereas CC2 forms a single continuous helix (Fig. 1*C*). CC proteins are known to be involved in many important biological functions, such as protein–protein interaction, dimerization/oligomerization, and gene expression (29, 30). To further characterize these domains, we expressed them as recombinant GST-tagged proteins in bacteria (Fig. 1*D*). Later, the GST tag was removed by digesting the GST-fusion proteins with thrombin treatment (Fig. 1*E*). The purified CC1 and CC2 domains were subjected to CD for secondary structural analysis. It revealed that CC1 and CC2 domains comprise 76.9% and 75.9% of α-helix, 13.7% and 14.9% of β-sheet (including parallel and antiparallel), and 8.10% and 8.2 % of the random coil, respectively, which is in close agreement with previous reports (29) (Figs. 1*F* and S1*B*).

As CC domains participate in protein–protein interactions, we employed a GST pull-down assay using GST-tagged CC1 and CC2 domains as baits and whole-cell protein extracts of MDA-MB231 as prey. The prominent proteins resolved on an SDS-PAGE were sliced out and analyzed by MALDI analysis. The four prominent proteins identified were myosin 9, isoform 2 of Krueppel-related zinc finger protein 1 (HKR1), CC domain–containing 105, and RUFY3 (Figs. 1, *G* and *H* and S1*C*). Earlier reports showed RUFY3 interacting with Rab5 as an early endosomal marker (31). However, the functional association among HPIP, RUFY3, and Rab5 is unknown. Despite Rab5 being a known interacting protein of RUFY3 (31), it was not present in the list of CC domain–interacting proteins possibly due to our limited and selective analysis of prominent but high-molecular-weight protein bands by MALDI, which has potentially eliminated Rab5 as it is a lower molecular weight protein (∼25 kDa).

### CC domains of HPIP are required for its interaction with RUFY3 and Rab5

**Figure 2:**
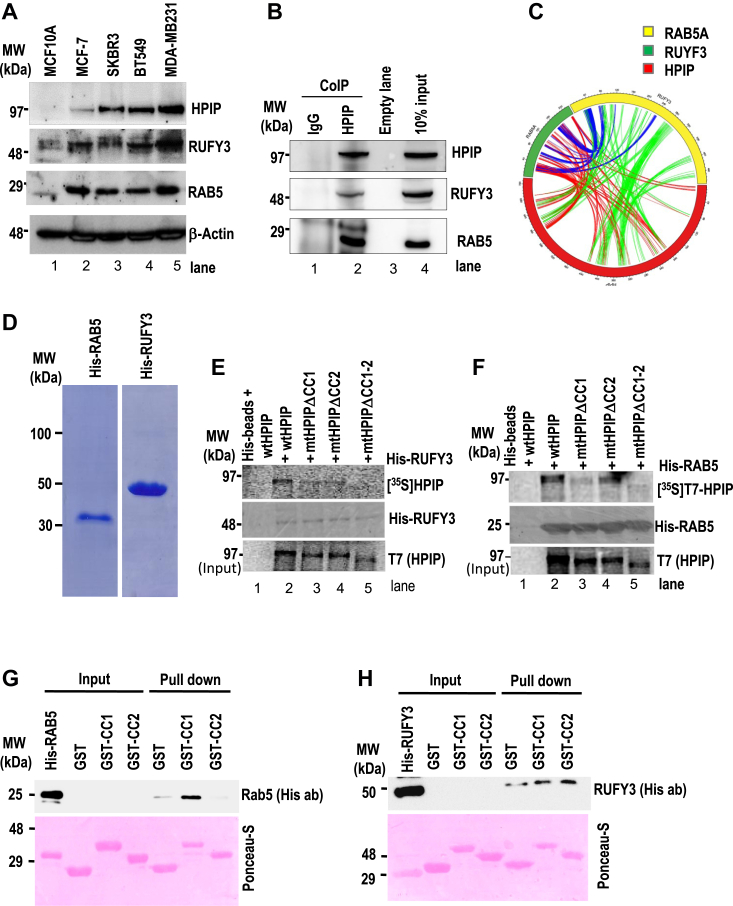
**Characterization of HPIP interaction with RUFY3 and Rab5.***A*, expression profile of HPIP, Rab5, and RUFY3 in a panel of breast cancer cells. *B*, coimmunoprecipitation (Co-IP) analysis demonstrating the *in vivo* interaction of HPIP with Rab5 and RUFY3 in MDA-MB231 cells. *C*, pair-wise contact predictions among three proteins RUFY3, Rab5, and HPIP, using Complex Contact circlize package (33) was shown in a circos diagram. *D*, SDS-PAGE separation of purified His-tagged RUFY3 and Rab5. Requirement of coiled coil domains of HPIP for its interaction with RUFY3 (*E*) or Rab5 (*F*) was demonstrated by *in vitro* pull-down assay. S^35^-Met–labeled wtHPIP, mtHPIPΔCC1, mtHPIPΔCC2, or mtHPIPΔCC1-2 were incubated with His-RUFY3 or His-Rab5 beads and after incubation at room temperature for 4 h, and followed by washing, protein complexes were analyzed by Western blotting as indicated. Conversely, GST-CC1 or GST-CC2 beads were incubated with purified His-Rab5 (*G*) or His-RUFY3 (*H*) at room temperature, and after washing, protein complexes were analyzed by Western blotting as indicated. CC, coiled-coil; HPIP, hematopoietic PBX–interacting protein; RUFY3, RUN FYVE domain–containing protein 3.

In order to characterize the functional association among HPIP, RUFY3, and Rab5, we first examined their expression in a panel of breast cancer cells. We found coexpression of HPIP, RUFY3, and Rab5 in most of the cell lines examined, while their relative abundance was more in MDA-MB231 cells and less in MCF10A cells (Fig. 2*A*). Subsequently, the coimmunoprecipitation (Co-IP) assay demonstrated their association in MDA-MB231 cells (Fig. 2*B*). Sequence-based coevolutionary signals are used to predict interfacial residues between two proteins (32). Using Complex Contact, we followed pair-wise contact predictions among three proteins, RUFY3, Rab5, and HPIP (33). We picked up the top 100 contacts predicted between each pair and constructed a complex interaction map among three proteins, as shown in Figure 2*C* as a circos diagram using the circlize package (34). We found a dense band of predicted interactions between 320 and 440 residues of HPIP and 272 to 296 and 392 to 408 residues of RUFY3. The same interaction region of HPIP is also shared for binding with residues from 24 to 38 of Rab5 protein. In addition, another thick band of interactions was found between 580 and 620 residue positions of HPIP and residue positions 125 to 130 and 210 to 215 regions of Rab5 (Fig. 2*C*). Next, we validated the requirement of CC domains in the interaction of HPIP with RUFY3 and Rab5. To check this, first, we generated HPIP mutants devoid of CC1, CC2 alone, or both domains such as mtHPIPΔCC1, mtHPIPΔCC2, and mtHPIPΔCC1-2, respectively, and subsequently, *in vitro* pull-down assay was performed using [S^35^]-labeled HPIP mutants and His-RUFY3 or His-Rab5 (Fig. 2*D*). The data revealed that RUFY3 could interact with WT HPIP only but not with mtHPIPΔCC1, mtHPIPΔCC2, or double mutant, that is, mtHPIPΔCC1-2, indicating that RUFY3 interacts with HPIP *via* CC1 and CC2 domains (Fig. 2*E*). Rab5 could interact with WT HPIP and mtHPIPΔCC2 but neither with mtHPIPΔCC1 nor mtHPIPΔCC1-2, indicating that Rab5 can interact with HPIP *via* the CC1 domain only (Fig. 2*F*). To support this data, *in vitro* GST pull-down assays were performed using GST-tagged CC1 or CC2 by incubating with purified His-tagged Rab5 or RUFY3. The data confirmed the involvement of CC1 and CC2 in interacting with RUFY3, albeit stronger affinity for CC2 than CC1, whereas CC1 alone interacts with Rab5 (Fig. 2, *G* and *H*). These results indicate that HPIP has two conserved CC domains, which present a unique architecture to the HPIP scaffolding function to directly interact with RUFY3 and Rab5, which may have a role in cellular signaling and function.

### HPIP and RUFY3 are noncanonical GEFs of Rab5

**Figure 3:**
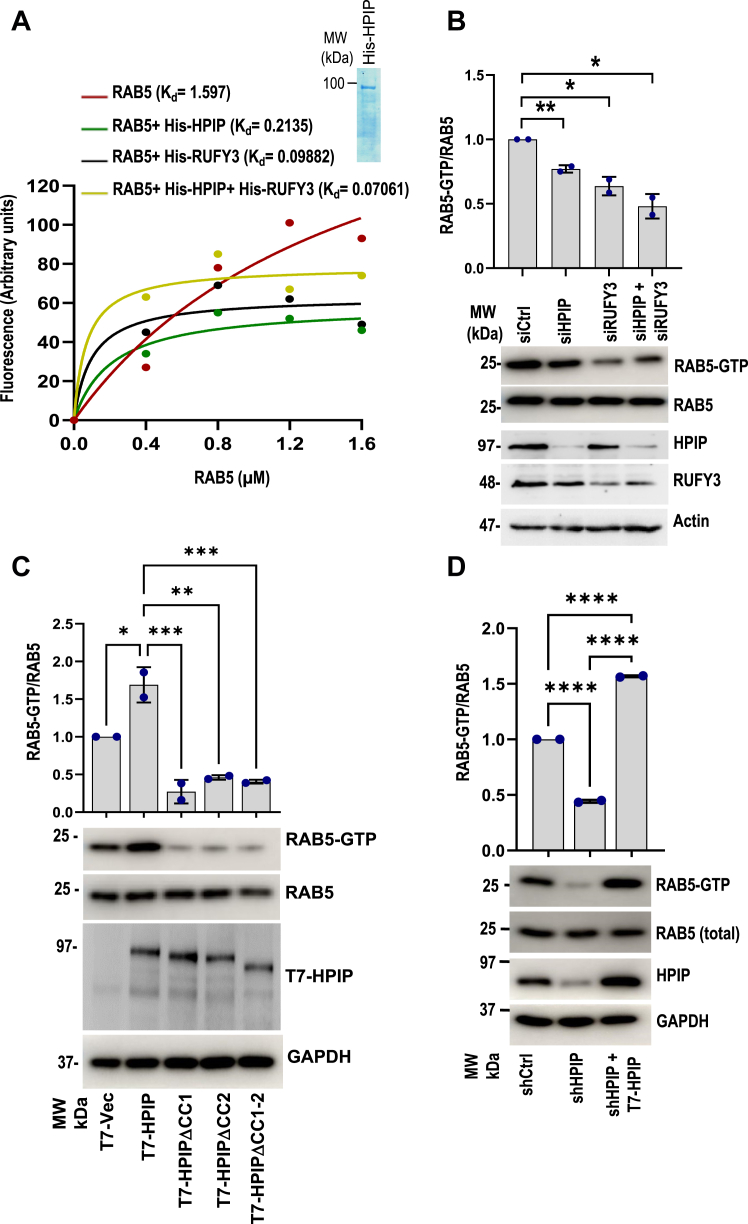
**HPIP and RUFY3 act as noncanonical guaninenucleotideexchange factors of Rab5.***A*, MAN-GTP–binding assay demonstrating GEF activities of HPIP, RUFY3 toward Rab5. Inset, SDS-PAGE separation of purified His-HPIP. *B*, MDA-MB231 cells were transiently transfected with siCtrl, siHPIP, siRUFY3, or siHPIP and siRUFY3. Forty-eight hours post transfection, cell extracts were subjected to Rab5 activation assay as described in the Methods and analyzed by Western blotting as indicated (*lower panel*). Quantification of Rab5 activation (*top panel*). *C*, Rab5 activation assay demonstrating the loss of Rab5 activation in CC mutants of HPIP. MDA-MB231 cells were transiently transfected with control pcDNA vector, wt-HPIP, mtHPIPΔCC1, mtHPIPΔCC2, or mtHPIPΔCC1-2, 48 h post transfection, cell extracts were subjected to Rab5 activation assay as described in the Methods and analyzed by Western blotting as indicated (*lower panel*). Quantification of Rab5 activation (*top panel*). *D*, cell extracts of MDA-MB231 cells stably transfected with either shCtrl or shHPIP and T7-HPIP (for rescue) were immunoprecipitated using active Rab5 antibody, which specifically binds to Rab5-GTP form, and analyzed by Western blotting as indicated. Error bars indicate SD. ∗*p* = <0.05; ∗∗*p* = <0.001. CC, coiled-coil; GEF, guanine nucleotide exchange factor; HPIP, hematopoietic PBX–interacting protein.

Activation of early endosomal protein Rab5 is crucial for endosome internalization and maturation, as ectopic expression of Rab5 (Q79L), a GTPase-defective mutant form of Rab5, results in the formation of enlarged early endosomes due to defective budding of the endosomal vesicles (35). Moreover, Rab5-GTP loading to endosomes is required for integrin internalization and, thus, cell migration (13). Given these earlier reports, we ascertained whether HPIP and RUFY3 act as GEFs of Rab5 by performing an *in vitro* MAN-GTP assay as described earlier (36). We cloned, bacterially expressed and purified recombinant proteins of HPIP, Rab5, and RUFY3 as His-tagged proteins (Fig. 3*A*) and then evaluated the GTP-binding potential of Rab5 by HPIP and RUFY3 as GEFs. Rab5 could bind to GTP with a dissociation constant (K_d_) of 1.59 ± 0.013 (Fig. 3*A*). The GTP binding of Rab5 was significantly elevated by incubating either recombinant His-HPIP (K_d_ = 0.21 ± 0.004) or His-RUFY3 (K_d_ = 0.098 ± 0.002) as compared to the control. Further elevation of Rab5 activation was also observed with combined incubation of HPIP and RUFY3, suggesting a synergistic effect on Rab5 activation (K_d_ = 0.07 ± 0.003) (Fig. 3*A*). Next, we evaluated Rab5 GEF functions of HPIP and RUFY3 in MDA-MB231 cells by analyzing GTP-bound Rab5 by probing cell extracts with Rab5-GTP antibody. Knockdown of HPIP or RUFY3 in MDA-MB231 cells significantly decreased GTP-bound Rab5 over control cells (Fig. 3*B*). Since CC domains of HPIP are crucial for its scaffolding activity, we next evaluated their role in Rab5 activation. Although wtHPIP could enhance the Rab5 activation over control cells, CC mutants of HPIP failed to do so (Fig. 3*C*). Next, we performed a rescue experiment by altering the expression of HPIP by the gain and loss-of-function approach. HPIP depletion significantly decreased GTP-bound Rab5 (active Rab5) over control cells (Fig. 3*D*, lane 2). Conversely, ectopic expression of T7-HPIP in HPIP-silenced cells restored Rab5 activation (Fig. 3*D*, lane 3). Together, these data suggested that HPIP and RUFY3 act as GEFs of Rab5.

### HPIP, Rab5, and RUFY3 localize to FAs as well as endosomes

**Figure 4:**
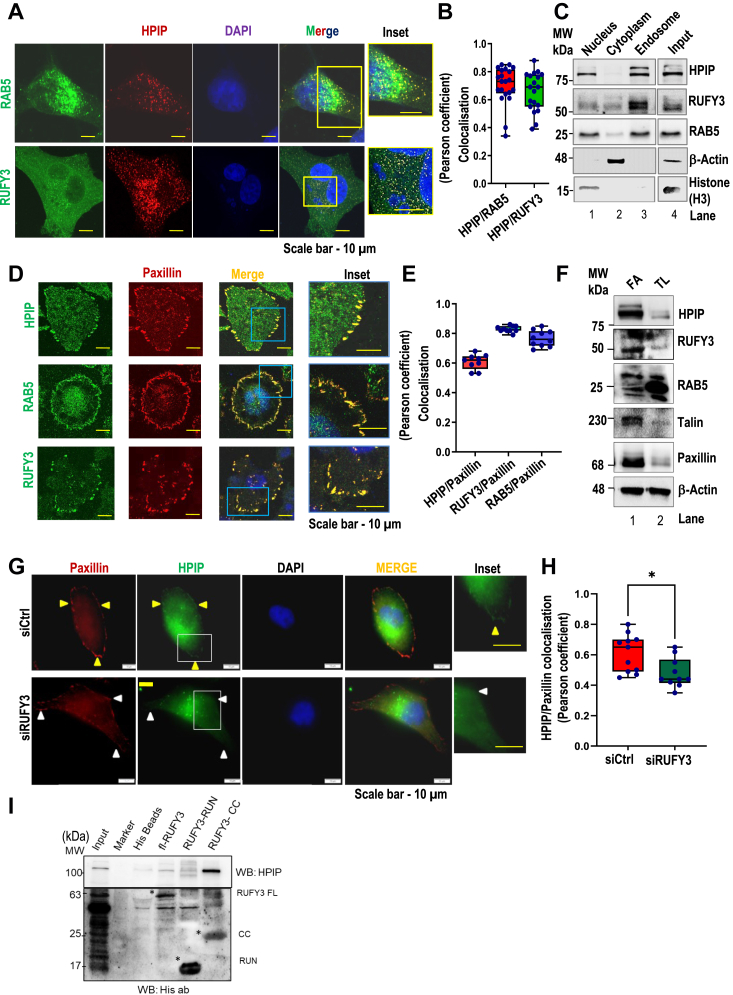
**Focal adhesions and endosome localization of HPIP, Rab5 and RUFY3 in MDA-MB-231 cells.***A*, confocal imaging demonstrates colocalization of HPIP with RUFY3 or Rab5 in MDA-MB231 cells. DAPI (*blue*); RUFY3 or Rab5 (*green*); HPIP (*red*). *B*, *E*, and *H*, colocalization of indicated proteins was quantified by Pearson correlation coefficient method of pixel intensity correlation measurements using Image J software. The scale bar represents 10 μm. Error bars indicate SD. *C*, MDA-MB231 cells were fractionated into cytoplasm, nucleus, and endosomes and subjected to Western blotting analysis by indicated antibodies. β-Actin, histone H3 and Rab5 were served as cytoplasmic, nucleus, and endosome markers, respectively. *D*, confocal imaging demonstrating colocalization of HPIP, RUFY3, or Rab5 (*green*) with Paxillin (*red*) in MDA-MB231 cells grown on fibronectin (FN)-coated plates. *F*, focal adhesion fractions (FA) isolated from MDA-MB231 cells were subjected to Western blotting analysis as indicated. Talin and Paxillin were served as focal adhesion markers. *G*, confocal imaging demonstrating RUFY3 depletion on HPIP localization to Paxillin-focal adhesion sites in MDA-MB231 cells. The scale bar represents 10 μm. *I*, *in vitro* pull down demonstrating the interaction of wt RUFY3 and RUFY3-CC domain but not RUFY3-RUN domain with HPIP. *Yellow arrowheads* denote localization of HPIP into focal adhesions (Paxillin) in shCtrl cells. *White arrowheads* denote inefficient localization of HPIP into focal adhesions in shRUFY3 cells. CC, coiled-coil; DAPI, 4′,6-diamidino-2-phenylindole; HPIP, hematopoietic PBX–interacting protein; RUFY3, RUN FYVE domain–containing protein 3.

After establishing the role of HPIP and RUFY3 as GEFs of Rab5, we next studied their subcellular localization to unravel the signaling mechanism and its associated function. Rab5 is a small GTPase that predominantly localizes to endosomes, as shown in Figure 4*A*. A punctate cytoplasmic distribution pattern for HPIP was observed with a significant colocalization with Rab5 (Pearson coefficient = 0.74 ± 0.01). Despite RUFY3 distributes throughout the cytoplasm, a significant colocalization with HPIP was observed in punctuate regions, which represents endosomes (Pearson coefficient = 0.69 ± 0.01) (Fig. 4, *A* and *B*). Further supporting this observation, biochemical fractionation analysis revealed marked enrichment of HPIP, RUFY3, and Rab5 in an endosomal fraction in MDA-MB231 cells (Fig. 4*C*).

It is well established that FN- induced integrin signaling regulates FA disassembly and cell migration (37). Given this, we examined the effect of FN on FA localization of HPIP, RUFY3, and Rab5 using Paxillin as a FA marker. As shown in Figure 4, *D* and *E*, a significant fraction of HPIP, RUFY3, and Rab5 are localized to FAs (Pearson coefficients for HPIP, RUFY3, and Rab5 with Paxillin are 0.61 ± 0.03, 0.82 ± 0.01 and 0.78 ± 0.01, respectively). Further, biochemical fractionation analysis showed a marked enrichment of HPIP, Rab5, and RUFY3 in FN-coated cells (Fig. 4*F*). Though earlier studies reported the FA localization of HPIP (27), the factors driving its FA localization are unknown. RUFY3 has been reported to regulate the cellular localization of small GTPase proteins such as Rap2 and TIAM2/STEF (38). Given the previous report, we ascertained whether RUFY3 recruits HPIP into FAs. Silencing of RUFY3 by RUFY3-specific siRNA indeed significantly decreased FA localization of HPIP, while increasing its cytoplasmic distribution in MDA-MB231 cells (Fig. 4, *G* and *H*). RUFY3 has three domains: CC, RUN, and FYVE. We next explored which domain of RUFY3 is required for its interaction with HPIP and, thus, possible role in FA localization. RUFY3 domains were cloned, expressed as His-tagged proteins, and used for pull-down assay. *In vitro,* interaction assay demonstrated the interaction of HPIP with the CC domain of RUFY3 but not with FYVE and RUN domains (Fig. 4*I*). Together these results indicate that HPIP, RUFY3, and Rab5 localize to FAs as well as endosomes, and RUFY3 recruits HPIP possibly *via* the CC domain into FAs.

### HPIP and RUFY3 are involved in Rab5-driven cell migration

**Figure 5:**
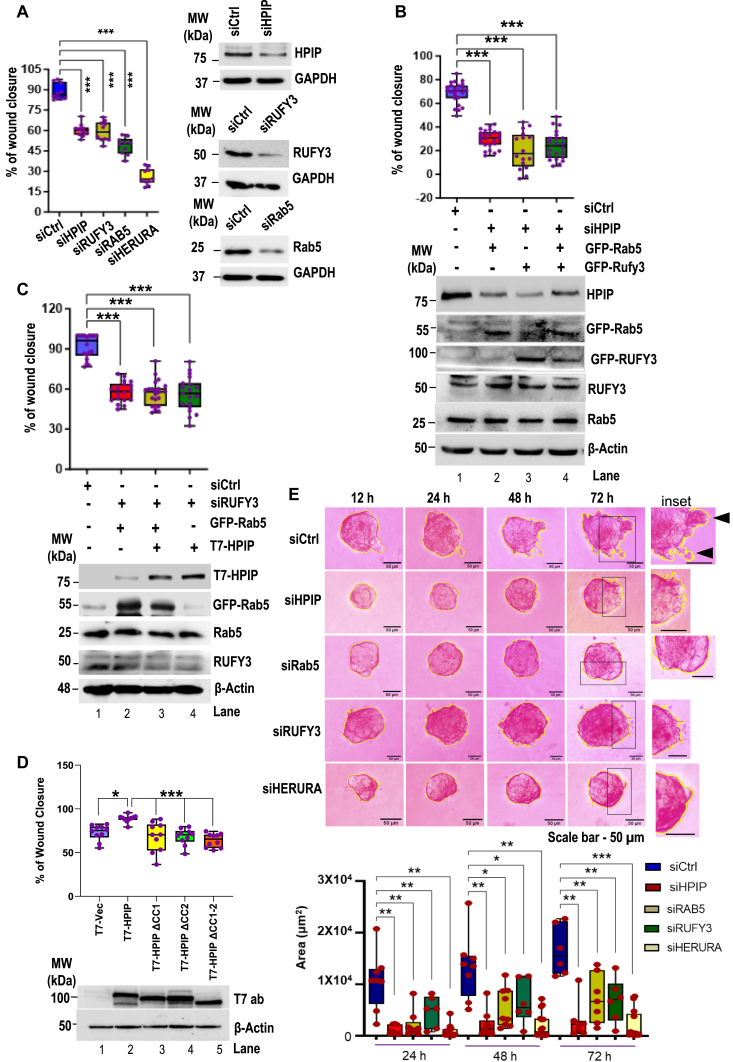
**HPIP and RUFY3 are required for Rab5-driven cell migration.***A*, scratch wound assay demonstrating the effect of siHPIP, siRUFY3, siRab5, or combined knockdown of these three proteins (siHERURA) on cell migration ability of MDA-MB231 cells (*left panel*). Western blot analysis showing knockdown of indicated proteins (*right panel*). Dependence of Rab5 on either HPIP (*B*) or RUFY3 (*C*) in cell migration ability of MDA-MB231 cells. Cells were cotransfected as indicated and cell migration was measured by scratch would assay (*top panel*). Western blot analysis showing the expression of various proteins as indicated (*bottom panel*). *D*, MDA-MB231 cells were transfected T7-vector, wtHPIP, mtHPIPΔCC1, mtHPIPΔCC2, or mtHPIPΔCC1-2 constructs and cell migration was measured by scratch wound assay (*top panel*). Western blot analysis showing the expression of wt HPIP and its CC domain mutants (*bottom panel*). *E*, hanging-drop cell invasion assay demonstrating the effect of siHPIP, siRUFY3, siRab5, or combined knockdown of these three proteins (siHERURA) on cell invasion ability of MDA-MB231 cells cultured on Matrigel. Cell periphery was marked with *yellow contour lines*. *Black arrowheads* in inset denotes cellular invasions in shCtrl cells. Error bars indicate SD. ∗∗∗*p* < 0.001, ∗∗*p* < 0.001 measured by Student’s *t* test. CC, coiled-coil; HPIP, hematopoietic PBX–interacting protein; RUFY3, RUN FYVE domain–containing protein 3.

After establishing the cellular localization of HPIP, Rab5, and RUFY3, we next focused on the functional significance of their interaction in cancer cells. Earlier independent studies showed the involvement of these proteins in cell migration (15, 27, 39). In light of these earlier reports, we ascertained that HPIP and RUFY3 might regulate cell migration *via* Rab5 activation. Knockdown of HPIP, Rab5, and RUFY3 alone or combined knockdown (HERURA, which represents HPIP, RUFY3, and Rab5) of these components significantly decreased cell migration of MDA-MB231 cells (Figs. 5*A* and S2*A*). We next performed rescue studies to check if Rab5-mediated cell migration depends on HPIP or RUFY3 expression. As shown in Figure 5*B* and Fig. S2*B*, cell migration was significantly hampered upon HPIP knockdown in MDA-MB231 cells, further over expression of Rab5, RUFY3, or both, however, failed to rescue it. In a similar experiment, over expression of HPIP, Rab5, or both failed to rescue MDA-MB231 cell migration in which RUFY3 was depleted (Figs. 5*C* and S2*C*). As we established that the interaction of HPIP with Rab5 and RUFY3 was dependent on CC domains in HPIP, we examined the cell migration ability of HPIP’s CC mutants. While wtHPIP could significantly enhance cell migration, as demonstrated by scratch wound closure assay, HPIP-CC mutants failed to do so (Figs. 5*D* and S2*D*). To further strengthen these findings, we performed cell invasion assays using 3D cultures of MDA-MB-231 cells grown in Matrigel. Silencing of HPIP, RUFY3, and Rab5 alone or combined knockdown (HERURA) significantly hampered the cell invasions measured by cellular protrusions (Fig. 5*E*). Together, these data support that HPIP–RUFY3–Rab5 axis modulate cell migration, which is partly dependent on the CC domains of HPIP.

### Rab5-mediated FA disassembly and FAK activation are dependent on GEF activities of HPIP and RUFY3

**Figure 6:**
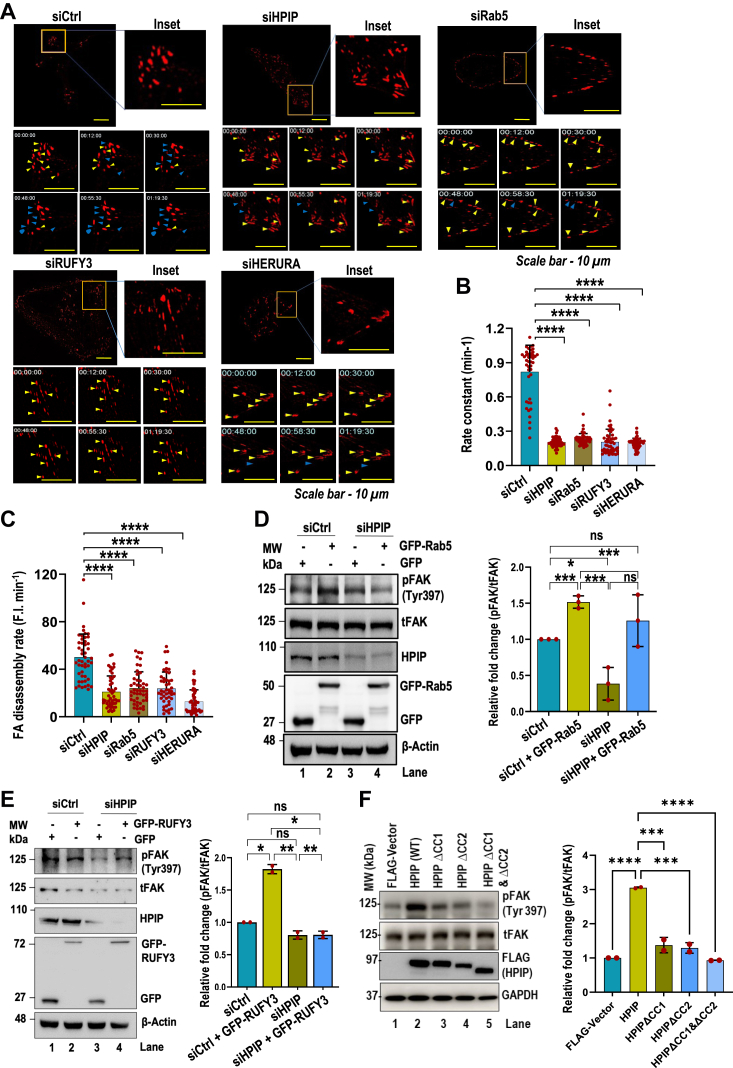
**HPIP and RUFY3 are required for Rab5-mediated focal adhesion disassembly.***A*, representative time lapse fluorescence images of MDA-MB231 cells expressing DsRed-Paxillin and control siRNA (siCtrl), siHPIP, siRUFY3, or siRab5 alone or together (siHERURA) at indicated time points. The scale bar represents 10 μm. Focal adhesion rate constant (min^−1^) (*B*) and disassembly rate (fluorescence intensity per min) (*C*) were calculated from Figure (*A*). *D*, FAK activation is demonstrated by Western blotting analysis of pFAK(Y397) in MDA-MB231 cells transfected with either siCtrl or siHPIP and ectopic expression of GFP-Rab5 (*left panel*). *E*, phosphorylation of FAK at Y397 in MDA-MB231 cells transfected with either siCtrl or siHPIP and ectopic expression of GFP-RUFY3 was analyzed by Western blotting. *F*, Western blot analysis of pFAK(Y397) in MDA-MB231 cells transfected with vector, wtHPIP, mtHPIPΔCC1, mtHPIPΔCC2, or mtHPIPΔCC1-2 constructs. Densitometric analysis of FAK activation in relative folds for (*D*, *E*, and *F*) (respective *right panels*). ∗∗∗*p* < 0.001, ∗∗*p* < 0.001 measured by Student’s *t* test. CC, coiled-coil; FAK, focal adhesion kinase; HPIP, hematopoietic PBX–interacting protein; RUFY3, RUN FYVE domain–containing protein 3.

We next focused on the cellular mechanism that underlies cell migration mediated by Rab5 in concert with HPIP and RUFY3. Earlier studies have demonstrated the role of HPIP and Rab5, but not RUFY3, in FA turnover (14, 27). Hence, we assessed if HPIP and RUFY3 could influence Rab5-mediated FA disassembly and FAK activation. We monitored the FA turnover upon silencing HPIP, Rab5, RUFY3, or combined knocked down (HERURA) in mCherry-Paxillin transfected MDA-MB231 cells seeded on FN-coated plates using high-resolution live-cell time lapse video microscopy. FA disassembly was monitored using mCherry-Paxillin as a FA tracking molecule and recorded in videos. Rate constants for net disassembly rates were generated from the plots of mCherry-Paxillin fluorescence intensities over time. We observed a significant decrease in rate constants and FA disassembly rate upon depletion of HPIP, RUFY3, or Rab5, implying that they regulate cell migration by controlling FA disassembly (Figs. 6, *A*–*C* and S3; Movies S1–S5). Even though Rab5 is known to promote cell migration *via* FAK activation, it is unclear whether Rab5-mediated activation of FAK is HPIP- or RUFY3-dependent. We examined FAK activation by altering the expression of HPIP, RUFY3, or Rab5. The data revealed that FAK activation by Rab5 is HPIP-dependent as HPIP depletion significantly abrogated it (Fig. 6*D*). Similar results were also observed for RUFY3, as it failed to activate FAK upon HPIP knockdown (Fig. 6*E*). Since HPIP bridges RUFY3 and Rab5, CC deletions in HPIP could also abolish FAK activation (Fig. 6*F*). Together, these results imply that Rab5-mediated FA disassembly *via* FAK activation is HPIP- and RUFY3-dependent.

### HPIP and RUFY3 are required for Rab5-mediated β1-integrin internalization and recycling

**Figure 7:**
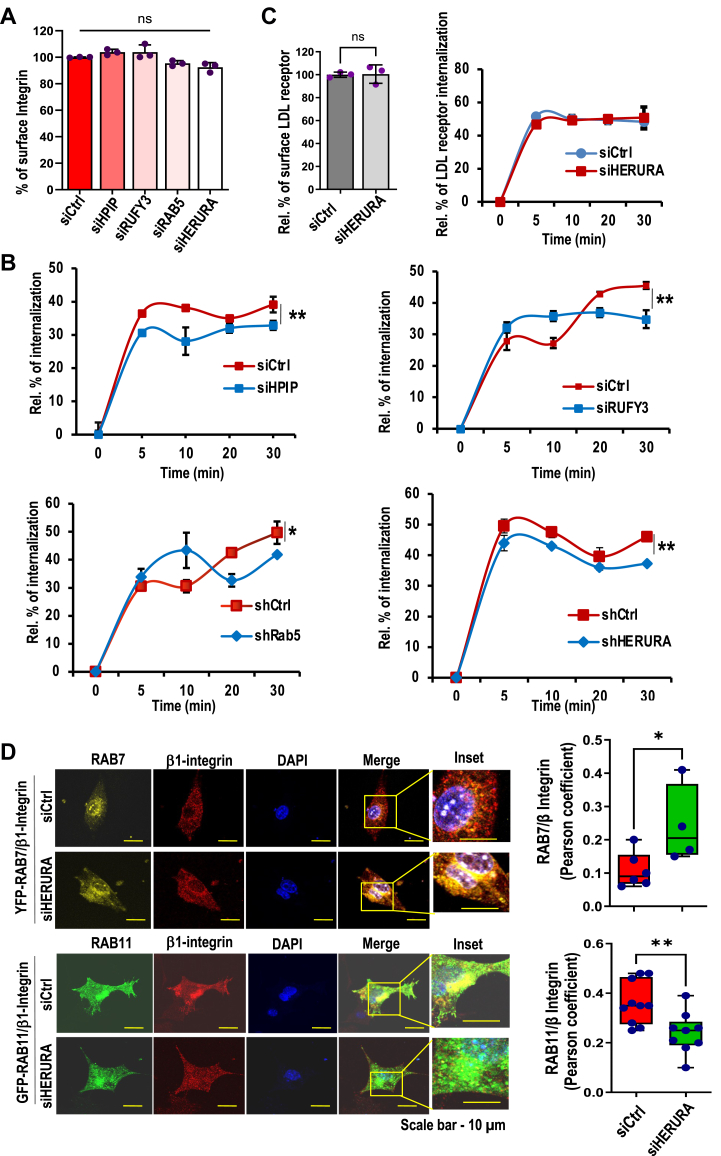
**Effect of HPIP or RUFY3 knockdown on Rab5-mediated β1-integrin internalization and endocytosis.***A*, percentage of surface β1-integrin in MDA-MB231 cells transfected with control siRNA (siCtrl), siHPIP, siRUFY3, siRab5 alone or together (siHERURA). *B*, biotin-labeled ELISA-based β1-integrin internalization assay demonstrating the percentage of internalized β1-integrin in MDA-MB231 cells transfected with control siRNA (siCtrl), siHPIP, siRUFY3, siRab5 alone or together (siHERURA) at indicated time points. Error bars indicate SD. ∗*p* = <0.05; ∗∗*p* = <0.001. *C*, under similar experimental conditions as mentioned in Figure (*B*), surface levels of LDL receptor and its internalization was measured. *D*, MDA-MB231 cells were cotransfected with siCtrl or siHERURA (siRNAs against HPIP, RUFY3, and Rab5) and YFP-Rab7 (*yellow*) construct (*top panel*) or GFP-Rab11 (*green*) construct (*lower panel*), and 36 h post transfection cells were analyzed by confocal imaging for colocalization of GFP-Rab7 or GFP-Rab11 with β1-integrin (*left panel*). Colocalization of indicated proteins was quantified by Pearson correlation coefficient method of pixel intensity correlation measurements using Image J software (*right panel*). Error bars indicate SD. The scale bar represents 10 μm. ∗*p* < 0.05 measured by Student’s *t* test. HPIP, hematopoietic PBX–interacting protein; ns, no significance; RUFY3, RUN FYVE domain–containing protein 3.

Earlier studies established that endocytosis regulates cell migration (1, 2); directional transport of integrins occurs in migrating cells (40). Furthermore, MTs and endocytosis are involved in the turnover of FAs (6, 8). Based on our microscopy studies demonstrating the colocalization of the HPIP, RUFY3, and Rab5 into FAs and endosomes, we hypothesized the possible involvement of these proteins in endocytosis-mediated cell migration. To test this possibility, we performed a MT regrowth assay using nocodazole washout experiments and measured integrin internalization as described previously (8). We followed β1-integrin, a FN receptor, internalization upon depletion of either individual components of three proteins by respective siRNAs or combined depletion (HERURA) in MDA-MB231 cells seeded on FN-coated plates. We observed a significant amount of β1-integrin localized to plasma membrane as well as cytoplasm in MDA-MB231 cells (Fig. S4, *A* and *B*). Depletion of individual components or together (HPIP, Rab5, and RUFY3) did not significantly alter the cell surface levels of total β1 integrin measured by ELISA-based assay (Fig. 7*A*). Conversely, the internalization rates of β1 integrin were significantly decreased in cells transfected with siRNAs for three proteins (Figs. 7*B* and S4*B*). Under similar experimental conditions, the internalization of LDL receptor was not altered, indicating the effect was specific to β1 integrin (Fig. 7*C*). The formation and disassembly of FAs during cell migration are coupled to integrins' recycling or lysosomal degradation *via* Rab proteins (1, 2). While GTPase Rab11 localizes to intracellular post Golgi membranes, including the *trans-*Golgi network and recycling endosomes, and participates in the recycling of endosomes, Rab7 has been ascribed to play a role in trafficking between late endosomes and lysosomes (1, 41, 42, 43). Given the earlier reports, we next checked whether HERURA is involved in recycling β1 integrin–associated endosomes. We first depleted HPIP, RUFY3, and Rab5 (HERURA) in MDA-MB231 cells by siRNA approach, followed by transfection of either YFP-Rab7 or GFP-Rab11 along with mCherry-β1 integrin. Then cells were treated with nocodazole (10 μM) for 4 h and released into fresh medium to ensure the MT regrowth and colocalization of these proteins were analyzed by confocal imaging. HPIP, RUFY3, and Rab5-depleted cells showed significantly increased colocalization of β1 integrin (red) endosomes with Rab7 (yellow) while decreased colocalization with Rab11 (green) as compared to control cells (Fig. 7*D*). Together these data suggest that the HPIP and RUFY3 are required for Rab5-mediated FN-associated β1-integrin internalization and recycling mechanism.

## Discussion

This study characterized HPIP and RUFY3 as novel GEFs of Rab5. Multiple lines of evidence suggest that HPIP and RUFY3 play critical roles in Rab5-mediated FA disassembly, FN-associated β1-integrin internalization, and recycling, which implicates in cell migration.

HPIP is a scaffolding protein. It is known to interact with several signaling proteins, including FAK, calpain2, PI3K, Src, and MTs that converge onto cell migration functions (17, 27). Even though our earlier study revealed that HPIP regulates cell migration *via* FAK activation, the underlying cellular mechanism remained elusive (27). Therefore, in this study, we further sought to identify novel interacting partners to provide new insights into HPIP-mediated cell migration. First, we identified two highly conserved CC domains in HPIP. CC domain is a versatile motif present in a plethora of proteins and is mainly involved in protein–protein interactions (44). Given this, we identified RUFY3 as a novel interacting partner of HPIP for the first-time using CC domains of HPIP as bait. Rab5 was not present in the list of CC domain–interacting proteins, possibly due to our limited and selective analysis of prominent but high-molecular-weight protein bands by MALDI that have potentially eliminated Rab5, as it is a lower molecular weight protein. RUFY3, also known as Singar1, was initially identified as a gene that suppresses the formation of surplus axons in neuronal polarity (45). Recent reports revealed its role in endoskeleton reorganization and cell migration (39, 46). RUFY3 is also known to act as an effector of Arl8b in controlling lysosome size and positioning (47). It acts as an adaptor protein for small GTPases, activating the Rac GTP/GDP exchange factor to control neural cell polarity (38). Interestingly, RUFY3 is known to interact with Rab5, however, the functional significance of this interaction was not explored (31). Keeping these earlier reports in view, we hypothesized HPIP and RUFY3 could influence Rab5 activation that may impart on cell migration. We successfully demonstrated that CC domains indeed bridge HPIP, Rab5, and RUFY3, forming a functional protein complex at FAs and endosomes.

Despite several independent reports documenting the role of Rab5, RUFY3, and HPIP in cell migration, the molecular interdependence of these proteins in the regulation of common cellular functions, such as cell migration, was unexplored. Using loss of function and rescue experiments, we demonstrate that HPIP and RUFY3 act as noncanonical GEFs of Rab5. FAK activation has been the nodal point for FA signaling and cell migration (48). Interestingly, both HPIP and Rab5 have been the activators of FAK (15, 27). However, it was intriguing to know whether Rab5-mediated FAK activation is HIPP-dependent. We provide evidence that HPIP is required for Rab5-mediated FAK activation. Because CC domains bridge three proteins, we observed impaired FAK activation by CC domain mutants of HPIP. Earlier studies have shown that FAK activation is required for FA disassembly and cell migration (8). Confocal imaging analysis demonstrated that HPIP, RUFY3, and Rab5 colocalize to FAs and regulate FA disassembly. Loss of expression of these three proteins severely impaired the FA disassembly rate and rate constants in MDA-MB231 cells. Together the results from these studies imply that Rab5-mediated FAK activation and FA dynamics are attributed to the Rab5 GEFs function of HPIP and RUFY3 in breast cancer cells.

We show that Rab5 regulates FA turnover by controlling integrin internalization and recycling. Integrin endocytosis is coupled with FA disassembly during cell migration (8). Interestingly, Rab proteins such as Rab5 and Rab11 are linked to integrin internalization and recycling in migrating cells (49, 50, 51). In support of these earlier arguments, few studies showed that proteins like caspase-8 promote Rab5-mediated internalization and recycling of β1 integrins by increasing Rab5 GTP loading through p85α interaction (13, 52). While Rab7 GTPase regulates late endosome trafficking, Rab11 is involved in the exocytosis of recycling vesicles (53, 54). Our data shows that FN triggers FA localization of HPIP, RUFY3, and Rab5 and drives β1-integrin internalization. Loss of expression of HPIP, RUFY3, or Rab5 resulted in reduced colocalization of β1-integrin with Rab11 while increased colocalization with Rab7, implying they are required for FN-induced β1-integrin internalization and FA disassembly, but not late endosome/lysosome–mediated degradation.

It has been demonstrated that the interaction of β1 integrins and Rab5 is dependent on GTP loading, and in turn, Rab5-GTP loading to endosomes appears crucial for normal and tumor cell migration (13). Rab5 becomes active upon binding to GTP, which is mediated by GEFs. Not only that but GEFs also control the localization of small GTPases (55). GEFs regulate Rab's activity by making contacts in the switch I and II regions near GTPase nucleotide–binding domain to facilitate nucleotide exchange. Rabex5 is the best-studied Vsp-9 domain containing GEF for Rab5 (56). Recent studies show Rabex5 controls its Rab5 GEF activity through allosteric interactions (57). Our GTP-binding studies demonstrated that HPIP and RUFY3 could enhance the GTP-binding activity of Rab5, indicating their GEF activity toward Rab5. Further, the deletion of CC domains in HPIP also led to reduced GTP binding by Rab5. Unlike the canonical GEFs, HPIP and RUFY3 do not possess Vsp-9 domains to instigate GTP binding by Rab5. Further studies are warranted to identify the key residues and domains in HPIP and RUFY3 in their role as noncanonical GEFs for Rab5.

In conclusion, we identified HPIP and RUFY3 as novel noncanonical GEFs of Rab5 that regulates FA-coupled endocytosis of β1-integrin. The FA junction is a hub of diverse signaling pathways and scaffold proteins such as HPIP that link these diverse cellular pathways. The importance of HPIP as a signaling scaffold at the FA points could not be denied owing to its rich amino acid diversity and the existence of CC domains, which act as recognition sites for many proteins. Owing to this very nature, overexpression of HPIP, which is indeed observed in several cancers (22, 27, 58, 59) could have a cascading effect on cellular motility. The triumvirate of HPIP, Rab5, and RUFY3 at the helm of molecular signaling in a cell could be a key determining factor for FA turnover and endocytosis, which implicates in cell migration.

## Experimental procedures

### Cell culture

MDA-MB231, MCF7, SKBR3, BT549, MCF10A, and HEK293T cells were obtained from the National Center for Cell Sciences. Except for MCF10A, all the cell lines were maintained at 37 °C with 5% CO_2_ in Dulbecco's modified Eagle's medium supplemented with 10% fetal bovine serum, 2 mM L-glutamine, 100 U/ml penicillin, and 100 μg/ml streptomycin. MCF10A cells were cultured in MEGM (mammary epithelial growth medium) (Lonza, CC-3150) supplemented with 5% Horse serum (Gibco), 100 U/ml penicillin, and 100 μg/ml streptomycin.

### Lentiviral transduction

Gene stable expression or knockdown in MDA-MB231 cells was carried out as described previously (23, 60). Cells were transfected with a gene-specific (HPIP, RUFY3, or Rab5) shRNAs (Thermo Fisher Scientific) along with packaging plasmids (pREV, VSV-G, and pΔR in the ratio of 1:0.4:0.5) using Lipofectamine 2000 (Invitrogen) in HEK293 cells. Forty-eight hours posttransfection, viral soups were collected and added to the cells. Subsequently, positive clones were selected by eliminating the untransfected cells using 1 μg/ml puromycin. After verifying the efficiency of gene knock down by Western blotting, we proceeded with these clones further for various studies. When necessary, cells were treated with viral soups carrying pMNDUS-HPIP, which express Flag-HPIP.

### Plasmid constructs and site-directed mutagenesis

CC1 and CC2 domains of HPIP were generated by PCR amplification using pcDNA3.1A-HPIP (full length) as a template using specific primers (Table S1). Amplified fragments were subcloned into the pGEX4T1 vector (GE Healthcare), and clones were verified by restriction digestion and sequencing. To study the importance of CC domains of HPIP, mutants with deleted CC of HPIP were generated by using pcDNA3.1-HPIP as a template. The HPIPΔCC1 mutant was generated by PCR amplifying the left (1–810 bps) and right (1023–2196 bps) DNA fragments of HPIP. These two fragments were ligated together at a similar EcoRV site, creating a mutant with 811 to 1022 bps deleted from the HPIP full-length gene. This mutant was cloned into the pcDNA3.1 vector, and clone confirmation was carried out using restriction digestion and DNA sequencing. Similarly, HPIPΔCC2, which is devoid of CC2 coding sequence from 1101 bp to 1243 bp, was subcloned into the pcDNA3.1 vector. Double deletion mutant, HPIPΔCC1-2, was generated using pcDNA3.1-HPIPΔCC1 and pcDNA3.1-HPIPΔCC2 as templates, and clone was confirmed by DNA sequencing. Using pBSKІІ-RUFY3 plasmid (a kind gift from Prof. Naoyuki Inagaki, Kagawa University) as a template, the *RUFY3* gene was amplified using primers listed in Table S1. The PCR fragments were subcloned into pDsRed-N1 vector (Clontech), and a clone was verified by restriction digestion. The full-length *RUFY3* gene was subcloned into the pET-28a vector through PCR amplification using the primers mentioned in Table S1. Full-length Rab5 was cloned into the pET-28a vector (Novagen) using specific primers (Table S1). Rab5 active mutant (Rab5Q79L) was generated by using pEGFP-Rab5 as a template through PCR amplification by replacing glutamine (Q) at position 79 with leucine (L) using the primers listed in Table S1 (61). The amplified plasmids were digested with the *Dpn*I enzyme, followed by transformation into *Escherichia coli* DH5α cells, and all clones were confirmed by DNA sequencing.

### Expression and purification of recombinant GST-tag and His-tag proteins

*E. coli* BL21 (DE3) cells harboring pGEX4T1-HPIP, pGEX4T1-HPIP-CC1, pGEX4T1-HPIP-CC2, pET-RUFY3, or pET-Rab5 were cultured and recombinant proteins were induced with IPTG (1 mM). After 3 h of induction, cells were harvested and resuspended in bacteria lysis buffer (50 mM Tris–HCl pH 8, glycerol 10%, glucose 20%, and protease inhibitor cocktail). The cell lysates were sonicated and separated by centrifugation at 11,000 rpm for 10 min at 4 °C, and the soluble fraction was collected. The supernatant was incubated with nickel beads for His-tag proteins or Glutathione Sepharose beads for GST-tag proteins (Clonetech) for 90 min at 4 °C, followed by three washes with Nonidet P-40 (NP 40) lysis buffer (50 mM Tris–HCl pH 8, glycerol 10%, NP 40 1%, and 137 mM NaCl and protease inhibitor cocktail). To check the protein purity, 20 to 30 μl of beads were loaded onto SDS-PAGE.

### GST pull-down assay

*In vitro,* transcription and translation of WT HPIP and CC deletion mutants of HPIP were performed using a TNT kit (Promega Scientific), where 1 μg template plasmid DNA was translated in the presence of (^35^S) methionine in a reaction volume of 50 μl. The GST pull-down assays were performed by incubating equal amounts of His-Rab5 or His-RUFY3 beads with 10 μl of *in vitro* translated (^35^S)-labeled protein in binding buffer (25 mM Tris–HCl, pH 8, 50 mM NaCl, 10% glycerol, 0.1% NP 40) for 90 min. Post incubation at 37 °C for 1 h, beads were washed five times with GST-binding buffer, followed by eluting the proteins using 2× SDS buffer and finally separating the proteins on SDS-PAGE. The interacted proteins were then visualized by autoradiography (Typhoon scanner).

### Dimer exchange assay

For the formation of heterodimer on the exchange between differently tagged proteins, His-Rab5 or His-RUFY3 and GST- HPIP-CC1, GST-HPIP-CC2, and GST-HPIP-CC1-2 were incubated together in a 1:2 M ratio at room temperature for 30 min in 500 μl volume of binding buffer (150 mM NaCl, 50 mM Tris buffer, pH 8). Samples were flash-frozen in liquid nitrogen to capture the resulting association state and then thawed on ice. After thoroughly washing the beads with binding buffer or NP40 buffer, the bound proteins were eluted by 2× SDS sample buffer and subjected to SDS-PAGE.

### Rab5 activation assay

Rab5 activation assay was performed according to the manufacturer protocols (Abcam or New East Biosciences). Forty-eight hours posttransfection of plasmid constructs or siRNA against the client proteins, cells were harvested, and 1 mg of protein extracts were immunoprecipitated using anti-Rab5(GTP) antibody for 1 h at 4 °C, followed by pulled down with AG beads and washed with 1× assay lysis buffer and were analyzed by Western blotting with respective antibodies.

### Cell migration assay

A wound closure assay was carried out to determine the cell migration as described previously (27). Cells grown in 60-mm dishes as confluent monolayers were mechanically scratched with a 20-μl pipette tip to create a wound. After washing the cells with PBS to remove cellular debris, wound closure was measured in pixels using bright field microscope. Images were captured under 10× magnifications using bright field microscope (Olympus) immediately after wound incision and at later time points. Apparently, 7 to 10 areas were measured in each experiment. Wound closure was converted into percentage and mean values were plotted in a graph.

### Immunoblotting and Co-IP

Immunoblotting and Co-IP analysis were performed as described previously (23, 27). Briefly, cells were lysed in RIPA lysis buffer (50 mM Tris-Cl, pH 7.4, 150 mM NaCl, 1% NP 40, 0.5% sodium deoxycholate, 1 mM PMSF, and 1× protease inhibitor cocktail) and subjected to SDS-PAGE, followed by Western blot using protein-specific antibodies mentioned in Table S2. For Co-IP, cells were lysed in NP-40 lysis buffer (20 mM Tris, pH 8, 137 mM NaCl, 10% glycerol, 1% NP 40, 2 mM EDTA, and 1× protease inhibitor cocktail). Immunoprecipitation with the indicated antibodies was carried out overnight at 4 °C and incubated with agarose A/G beads for 1 h. After thorough washing, protein complexes were dissolved in 2× SDS loading dye and then subjected to Western blot.

### MANT-GTP assay

The MANT-GTP assay was performed using a previously described protocol (36). Briefly summarize, GST-Rab5–bound glutathione Sepharose beads were treated with a buffer (50 mM Tris–HCl pH 7.4, 150 mM NaCl, 1 mM DTT, 10 mM EDTA pH 8) at 4 °C for 30 min to remove the bound nucleotides. PreScission protease (GE Healthcare, 27-0843-01) was used to cleave GST overnight at 4 °C in a buffer (50 mM Tris–HCl pH 7.4, 150 mM NaCl, 1 mM DTT, 10 mM MgCl_2_), and the resulting cleaved Rab5 was collected. This protein was then loaded onto a glutathione Sepharose HR16/10 column, and unbound proteins were buffer exchanged into Hepes-buffered saline (25 mM Hepes 7.4, 150 mM NaCl, 10 mM MgCl2). The protein concentration was determined using a Lowry assay (Sigma-Aldrich). To conduct the MANT-GTP assay, various concentrations of Rab5 (0.4 μM, 0.8 μM, 1.2 μM, and 1.6 μM) were mixed with either His-RUFY3 (1 μM), His-HPIP (1 μM), or both HPIP/RUFY3, along with MANT-GTP (750 μM) (Molecular Probes, Cat# M-12415). The resulting mixture was incubated in a buffer (25 mM Hepes 7.4, 150 mM NaCl, 10 mM MgCl2) to a final volume of 200 μl for 2 h at 30 °C in a black-walled 96-well plate. Fluorescence measurements were taken by subtracting the fluorescence at time 0 from the fluorescence at each time point (Ex 355 nm; Em 449 nm; cut-off 435 nm). Data were analyzed by nonlinear regression using GraphPad Prism for curve fitting and K_d_ (dissociation constant) calculations.

### Hanging drop cell invasion assay

Briefly, 20 μl of MDA-MB-231 cells (siHPIP, siRab5, siRUFY3, siHERURA) consisting of 1000 cells were grown as hanging drops culture in Petri dishes by inverting the lid for 48 h with proper humidification inside the incubator. In prechilled 96 well-plates, 200 μl of Matrigel was poured and above that hanging-drop spheroids were carefully embedded. The cellular dissemination was monitored by capturing images at 0 h, 12 h, 24 h, 42 h, and 78 h in the mentioned siRNA-treated cells using a bright field microscope (Model IX81, Olympus). Using Fiji/ImageJ (https://imagej.net/ij/), the outgrowth area was determined in each well by subtracting the area at 0 h from the area of other time points.

### Microscopy and live imaging analysis

Cells cultured on either normal or FN-coated coverslips were fixed with 4% paraformaldehyde and permeabilized by prechilled acetone and methanol (1:3), as previously described (27). Primary antibodies at 1:100 dilutions were added to the cells. After overnight incubation at 4 °C, cells were treated with fluorescent-labeled secondary antibodies for 1 h. Fluorescent images were captured by confocal microscope (Model - NLO710, Carl Zeiss). Pearson colocalization coefficient was used to measure the degree of colocalization of two proteins using Image J. To determine the FA dynamics, cells were cotransfected with gene-specific siRNAs and DsRed-Paxillin using Lipofectamine 2000. After 48 h of transfection, the dynamics of DsRed-Paxillin at FA points were monitored for 1 h on a fluorescence microscope (Model -IX83, Olympus; inverted microscope with Oko Lab Uno live-cell chamber and Retiga 6000 monochrome detector). As described previously, FA dynamics quantification was performed (62). Rate constant measurements for the disassembly of FAs were determined from the slopes of trend lines fitted to semilogarithmic plots of fluorescence intensity ratios over time, as described previously (62).

### Biochemical fractionation and FA isolation

Subcellular fractionation was performed as described previously (63). Briefly, adherent cells were trypsinized and washed twice with PBS. Cells were incubated in 1 ml of the hypotonic lysis buffer (10 mM Hepes-KOH pH 7.2, 0.25 M Sucrose, 1 mM EDTA, 1 mM MgoAc, and protease and phosphatase inhibitors). Cell lysis was performed using the Dounce homogenizer, and nuclei were removed with 10 min 1000*g* centrifugation. The plasma membrane fraction was collected with 10 min 10000*g* centrifugation. The endosomal and cytoplasmic fractions were collected with 1 h 100,000*g* centrifugation. All fractionation experiments were performed at 4 °C. The pellets were dissolved in a sample buffer for Western blotting.

FA was isolated, as described previously (64). MDA-MB231 cells grown on FN-coated plates were incubated in triethanolamine-containing low ionic strength buffer (2.5 mM triethanolamine, pH 7), for 2 to 3 min at room temperature to hypnotically shock the cells and weaken cell membrane integrity. Intense and pulsed hydrodynamic forces were applied to the cells, with the PBS buffer and protease inhibitors, using the Oracura table-top water flosser. All cell bodies, membrane-bound organelles, nuclei, cytoskeleton, and soluble cytoplasmic components were removed after applying this force, leaving only the FA adhered to the tissue culture plate. These FA were then collected using 1× RIPA with 0.1% SDS and evaluated using Western blotting.

### Biotin-based integrin internalization assay

Biotin-based integrin internalization assays were performed as described previously (65). MDA-MB-231 (80% confluence) were serum starved for 30 min, washed twice in cold PBS, followed by surface labeling of NHS-SS-biotin (0.2 mg/ml) (Thermo Fischer Scientific) in PBS for 30 min at 4 °C. Labeled cells were washed with cold PBS to remove unbound biotin, and the biotin-labeled surface proteins were allowed to internalize by incubating with prewarmed 10% serum-containing Dulbecco's modified Eagle's medium at 37 °C in the presence of 0.6 μM primaquine (Abcam). One dish of cells was kept on ice to use for the detection of total surface biotinylation (without the treatment of MesNa). The medium was aspirated at the indicated time points, and the dishes were quickly transferred back to ice and washed twice with ice-cold PBS. The remaining biotin after internalization was removed from the cell’s surface by incubating with a solution containing 60 mM MesNa in 50 mM Tris (pH 8.6) and 100 mM NaCl for 30 min at 4 °C, followed by quenching of MesNa with 20 mM Iodoacetamide (Abcam) for 10 min on ice. Cells were washed with PBS and lysed in 200 mM NaCl, 75 mM Tris, 15 mM NaF, 1.5 mM Na3VO4, 7.5 mM EDTA, 7.5 mM EGTA, 1.5% Triton X-100, 0.75% IGEPAL CA-630, 50 g/ml leupeptin, 50 g/ml aprotinin, and 1 mM 4-(2-aminoethyl) benzenesulfonyl fluoride and incubated at 4 °C for 20 min. To determine the amount of internalized, biotinylated integrins, a 96-well plate (overnight coated with 5 μg/ml anti-β1 integrin antibody in 0.05 M Na_2_CO_3_, pH 9.6 at 4 °C) was blocked in PBS containing 0.05% Tween 20 (PBS-T) with 5% BSA for 1 h at room temperature, followed by the overnight incubation of the cell lysate (equal amount of protein) at 4 °C. After extensive washing with PBS-T to remove unbound materials, wells were incubated with streptavidin-conjugated horseradish peroxidase (Abcam) in PBS-T containing 1% BSA for 1 h at 4 °C. Wells were washed five to ten times with PBS-T and biotinylated integrins were detected by chromogenic reaction, incubation in dark with 100 μl of substrate solution prepared by dissolving *ortho-*phenylenediamine (0.4 mg/ml) in 0.05 M phosphate-citrate buffer, pH 5 with 30% hydrogen peroxide for 30 min in room temperature. The chromogenic reaction was stopped by adding 50 μl of 2N H_2_SO_4_ (stop solution) reading the absorbance at 450 nm (without stop solution) or 492 nm (with stop solution).

### Integrin internalization assay (subcellular fractionation by ultracentrifugation)

MDA-MB-231 cells were transfected either with siCtrl or siHPIP, siRAB5A, and siRUFY3. After a period of 36 h post transfection, cells were supplemented with growth factors deprived medium for 1 h. Cells were treated with complete medium for different time intervals (0, 5, 15, and 30 min). Afterward, cells were scraped in subcellular fractionation buffer (250 mM Sucrose, 20 mM Hepes, 10 mM KCl, 1.5 mM MgCl_2_, 1 mM EDTA, 1 mM EGTA, 1 mM DTT, and supplemented with protease inhibitor cocktail). Cell suspension was collected in a microcentrifuge tube and subjected to mechanical disruption by passing it through a 25 Gauge (G) syringe for ten times, 30 μl suspension taken out as direct lysate. Disrupted cells were centrifuged at 3000 rpm for 10 min, supernatant fraction was carefully collected in a fresh tube, and the pellet containing nuclear fraction was stored for further analysis. To obtain, membrane fraction, the supernatant was centrifuged at 100,000*g* for 1 h at 4 °C. After centrifugation, pellet was resuspended in 400 μl of subcellular fractionation buffer, disrupted by passing through 25 G syringe ten times and recentrifuged at 1,00,000*g* for 45 min at 4 °C. After discarding the supernatant, pellet was reconstituted in lysis buffer (50 mM Tris–HCl, 1 % Nonidet 40, 150 mM NaCl, 0.1 % SDS, 0.5 % sodium deoxycholate, 10 % glycerol, and protease inhibitor cocktail) and used for Western blot analysis.

### CD analysis

CD spectra of CC domains were recorded with a spectropolarimeter (Model: Jasco J-810), using a quartz cell with a path length of 0.02 cm. Five scans were run at a scan speed of 50 nm min^−1^, while data was collected at every 1 nm from 190 to 300 nm. An ellipticity of CD spectra is expressed in millidegrees. The protein secondary structure was constructed by CDNN 2.1 software (https://cdnn.software.informer.com/2.1/).

### Bioinformatics and protein structure modeling

CC domains for HPIP were identified by running the Coil server (www.expasy.com/coil) using the HPIP protein sequence. The structure for CC domains was determined by I-TASSER software (https://zhanggroup.org/I-TASSER/) (66, 67, 68). CC1 of HPIP was modeled using 3o0Z. A template had 34% sequence similarity and 79% sequence coverage. CC2 of HPIP was modeled 5vr2.2. As a template, with 35% sequence similarity and 98% sequence coverage. Sequences of the two CC elements were individually used for template selection and model building using the Swiss model server (69). We have done pairwise contact predictions between the three proteins, RUFY3, RAB5A, and HPIP, using Complex Contact as a circus diagram drawn using a circlize package (33, 34).

### Statistical analysis

The results are expressed as means ± SD, and differences between groups were analyzed by one-way ANOVA using either Sigma plot or GraphPad Prism. All the *p* values were calculated using the Student’s *t*-test using Sigma Plot or GraphPad Prism.

## Data availability

All data are contained within the manuscript. All the original Western blotting data will be shared upon request and may be contacted by first authors, S. S. K. (khsaratsingh@gmail.com) or V. P. (vasu2168@gmail.com).

## Supporting information

This article contains Supporting information.

## Conflict of interest

The authors declare that they have no conflicts of interest with the contents of this article.

## References

[1] Jones M.C., Caswell P.T., Norman J.C. (2006). Endocytic recycling pathways: emerging regulators of cell migration. Curr. Opin. Cell Biol..

[2] Paul N.R., Jacquemet G., Caswell P.T. (2015). Endocytic trafficking of integrins in cell migration current biology. Curr. Biol..

[3] Lawson M.A., Maxfield F.R. (1995). Ca(2+)- and calcineurin-dependent recycling of an integrin to the front migrating neutrophils. Nature.

[4] Palecek S.P., Schmidt C.E., Lauffenburger D.A., Horwitz A.F. (1996). Integrin dynamics on the tail region of migrating fibroblasts. J. Cell Sci..

[5] West M.A., Bretscher M.S., Watts C. (1989). Distinct endocytotic pathways in epidermal growth factor-stimulated human carcinoma A431 cells the. J. Cell Biol..

[6] Ezratty E.J., Bertaux C., Marcantonio E.E., Gundersen G.G. (2009). Clathrin mediates integrin endocytosis for focal adhesion disassembly in migrating cells. J. Cell Biol..

[7] Webb D.J., Parsons J.T., Horwitz A.F. (2002). Adhesion assembly, disassembly and turnover in migrating cells -- over and over and over again. Nat. Cell Biol..

[8] Ezratty E.J., Partridge M.A., Gundersen G.G. (2005). Microtubule-induced focal adhesion disassembly is mediated by dynamin and focal adhesion kinase. Nat. Cell Biol..

[9] Michael K.E., Dumbauld D.W., Burns K.L., Hanks S.K., Garcia A.J. (2009). Focal adhesion kinase modulates cell adhesion strengthening via integrin activation. Mol. Biol. Cell.

[10] Zerial M., McBride H. (2001). Rab proteins as membrane organizers. Nat. Rev. Mol. Cell Biol..

[11] Zhen Y., Stenmark H. (2015). Cellular functions of Rab GTPases at a glance. J. Cell Sci..

[12] Lanzetti L., Palamidessi A., Areces L., Scita G., Di Fiore P.P. (2004). Rab5 is a signalling GTPase involved in actin remodelling by receptor tyrosine kinases. Nature.

[13] Torres V.A., Mielgo A., Barbero S., Hsiao R., Wilkins J.A., Stupack D.G. (2010). Rab5 mediates caspase-8-promoted cell motility and metastasis. Mol. Biol. Cell.

[14] Torres V.A., Stupack D.G. (2011). Rab5 in the regulation of cell motility and invasion. Curr. Protein Pept. Sci..

[15] Mendoza P., Ortiz R., Diaz J., Quest A.F., Leyton L., Stupack D. (2013). Rab5 activation promotes focal adhesion disassembly, migration and invasiveness in tumor cells. J. Cell Sci..

[16] Mai H., Xu X., Mei G., Hong T., Huang J., Wang T. (2016). The interplay between HPIP and casein kinase 1alpha promotes renal cell carcinoma growth and metastasis via activation of mTOR pathway. Oncogenesis.

[17] Manavathi B., Acconcia F., Rayala S.K., Kumar R. (2006). An inherent role of microtubule network in the action of nuclear receptor. Proc. Natl. Acad. Sci. U. S. A..

[18] Shostak K., Patrascu F., Goktuna S.I., Close P., Borgs L., Nguyen L. (2014). MDM2 restrains estrogen-mediated AKT activation by promoting TBK1-dependent HPIP degradation. Cell Death Differ..

[19] Xu X., Fan Z., Kang L., Han J., Jiang C., Zheng X. (2013). Hepatitis B virus X protein represses miRNA-148a to enhance tumorigenesis. J. Clin. Invest..

[20] Khumukcham S.S., Penugurti V., Soni A., Uppala V., Hari K., Jolly M.K. (2022). A reciprocal feedback loop between HIF-1alpha and HPIP controls phenotypic plasticity in breast cancer cells. Cancer Lett..

[21] Penugurti V., Khumukcham S.S., Padala C., Dwivedi A., Kamireddy K.R., Mukta S. (2021). HPIP protooncogene differentially regulates metabolic adaptation and cell fate in breast cancer cells under glucose stress via AMPK and RNF2 dependent pathways. Cancer Lett..

[22] Bugide S., Gonugunta V.K., Penugurti V., Malisetty V.L., Vadlamudi R.K., Manavathi B. (2017). HPIP promotes epithelial-mesenchymal transition and cisplatin resistance in ovarian cancer cells through PI3K/AKT pathway activation. Cell. Oncol. (Dordr.).

[23] Khumukcham S.S., Samanthapudi V.S.K., Penugurti V., Kumari A., Kesavan P.S., Velatooru L.R. (2019). Hematopoietic PBX-interacting protein is a substrate and an inhibitor of the APC/C-CDC20 complex and regulates mitosis by stabilizing cyclin B1. J. Biol. Chem..

[24] Dwivedi A., Padala C., Kumari A., Khumukcham S.S., Penugurti V., Ghosh S. (2022). Hematopoietic PBX-interacting protein is a novel regulator of mammary epithelial cell differentiation. FEBS J..

[25] Khumukcham S.S., Manavathi B. (2021). Two decades of a protooncogene HPIP/PBXIP1: uncovering the tale from germ cell to cancer. Biochim. Biophys. Acta Rev. Cancer.

[26] Ji Q., Xu X., Kang L., Xu Y., Xiao J., Goodman S.B. (2019). Hematopoietic PBX-interacting protein mediates cartilage degeneration during the pathogenesis of osteoarthritis. Nat. Commun..

[27] Bugide S., David D., Nair A., Kannan N., Samanthapudi V.S., Prabhakar J. (2015). Hematopoietic PBX-interacting protein (HPIP) is over expressed in breast infiltrative ductal carcinoma and regulates cell adhesion and migration through modulation of focal adhesion dynamics. Oncogene.

[28] Abramovich C., Chavez E.A., Lansdorp P.M., Humphries R.K. (2002). Functional characterization of multiple domains involved in the subcellular localization of the hematopoietic Pbx interacting protein (HPIP). Oncogene.

[29] Liu J., Zheng Q., Deng Y., Cheng C.S., Kallenbach N.R., Lu M. (2006). A seven-helix coiled coil. Proc. Natl. Acad. Sci. U. S. A..

[30] Rose A., Meier I. (2004). Scaffolds, levers, rods and springs: diverse cellular functions of long coiled-coil proteins cellular and molecular life sciences. Cell. Mol. Life Sci..

[31] Yoshida H., Okumura N., Kitagishi Y., Shirafuji N., Matsuda S. (2010). Rab5(Q79L) interacts with the carboxyl terminus of RUFY3. Int. J. Biol. Sci..

[32] Hopf T.A., Scharfe C.P., Rodrigues J.P., Green A.G., Kohlbacher O., Sander C. (2014). Sequence co-evolution gives 3D contacts and structures of protein complexes. Elife.

[33] Zeng H., Wang S., Zhou T., Zhao F., Li X., Wu Q. (2018). ComplexContact: a web server for inter-protein contact prediction using deep learning. Nucleic Acids Res..

[34] Gu Z., Gu L., Eils R., Schlesner M., Brors B. (2014). Circlize Implements and enhances circular visualization in R. Bioinformatics.

[35] Ceresa B.P., Lotscher M., Schmid S.L. (2001). Receptor and membrane recycling can occur with unaltered efficiency despite dramatic Rab5(q79l)-induced changes in endosome geometry the. J. Biol. Chem..

[36] Whitecross D.E., Anderson D.H. (2017). Identification of the binding sites on Rab5 and p110beta phosphatidylinositol 3-kinase. Sci. Rep..

[37] Nagano M., Hoshino D., Koshikawa N., Akizawa T., Seiki M. (2012). Turnover of focal adhesions and cancer cell migration. Int. J. Cell Biol..

[38] Honda A., Usui H., Sakimura K., Igarashi M. (2017). Rufy3 is an adapter protein for small GTPases that activates a Rac guanine nucleotide exchange factor to control neuronal polarity the. J. Biol. Chem..

[39] Wang G., Zhang Q., Song Y., Wang X., Guo Q., Zhang J. (2015). PAK1 regulates RUFY3-mediated gastric cancer cell migration and invasion. Cell Death Dis..

[40] Paul N.R., Allen J.L., Chapman A., Morlan-Mairal M., Zindy E., Jacquemet G. (2015). alpha5beta1 integrin recycling promotes Arp2/3-independent cancer cell invasion via the formin FHOD3 the. J. Cell Biol..

[41] Chavrier P., Parton R.G., Hauri H.P., Simons K., Zerial M. (1990). Localization of low molecular weight GTP binding proteins to exocytic and endocytic compartments. Cell.

[42] Vanlandingham P.A., Ceresa B.P. (2009). Rab7 regulates late endocytic trafficking downstream of multivesicular body biogenesis and cargo sequestration the. J. Biol. Chem..

[43] Welz T., Wellbourne-Wood J., Kerkhoff E. (2014). Orchestration of cell surface proteins by Rab11. Trends Cell Biol..

[44] Truebestein L., Leonard T.A. (2016). Coiled-coils: the long and short of it. Bioessays.

[45] Mori T., Wada T., Suzuki T., Kubota Y., Inagaki N. (2007). Singar1, a novel RUN domain-containing protein, suppresses formation of surplus axons for neuronal polarity the. J. Biol. Chem..

[46] Wei Z., Sun M., Liu X., Zhang J., Jin Y. (2014). Rufy3, a protein specifically expressed in neurons, interacts with actin-bundling protein Fascin to control the growth of axons. J. Neurochem..

[47] Kumar G., Chawla P., Dhiman N., Chadha S., Sharma S., Sethi K. (2022). RUFY3 links Arl8b and JIP4-Dynein complex to regulate lysosome size and positioning. Nat. Commun..

[48] Hamadi A., Bouali M., Dontenwill M., Stoeckel H., Takeda K., Ronde P. (2005). Regulation of focal adhesion dynamics and disassembly by phosphorylation of FAK at tyrosine 397. J. Cell Sci..

[49] Cheng K.W., Lahad J.P., Kuo W.L., Lapuk A., Yamada K., Auersperg N. (2004). The RAB25 small GTPase determines aggressiveness of ovarian and breast cancers. Nat. Med..

[50] Stenmark H. (2009). Rab GTPases as coordinators of vesicle traffic. Nat. Rev. Mol. Cell Biol..

[51] Subramani D., Alahari S.K. (2010). Integrin-mediated function of Rab GTPases in cancer progression. Mol. Cancer.

[52] Torres V.A., Mielgo A., Barila D., Anderson D.H., Stupack D. (2008). Caspase 8 promotes peripheral localization and activation of Rab5. J. Biol. Chem..

[53] Rink J., Ghigo E., Kalaidzidis Y., Zerial M. (2005). Rab conversion as a mechanism of progression from early to late endosomes. Cell.

[54] Takahashi S., Kubo K., Waguri S., Yabashi A., Shin H.W., Katoh Y. (2012). Rab11 regulates exocytosis of recycling vesicles at the plasma membrane. J. Cell Sci..

[55] Cherfils J., Zeghouf M. (2013). Regulation of small GTPases by GEFs, GAPs, and GDIs. Physiol. Rev..

[56] Horiuchi H., Lippe R., McBride H.M., Rubino M., Woodman P., Stenmark H. (1997). A novel Rab5 GDP/GTP exchange factor complexed to Rabaptin-5 links nucleotide exchange to effector recruitment and function. Cell.

[57] Lauer J., Segeletz S., Cezanne A., Guaitoli G., Raimondi F., Gentzel M. (2019). Auto-regulation of Rab5 GEF activity in Rabex5 by allosteric structural changes, catalytic core dynamics and ubiquitin binding. Elife.

[58] van Vuurden D.G., Aronica E., Hulleman E., Wedekind L.E., Biesmans D., Malekzadeh A. (2014). Pre-B-cell leukemia homeobox interacting protein 1 is overexpressed in astrocytoma and promotes tumor cell growth and migration. Neuro Oncol..

[59] Xu X., Jiang C., Wang S., Tai Y., Wang T., Kang L. (2013). HPIP is upregulated in liver cancer and promotes hepatoma cell proliferation via activation of G2/M transition. IUBMB life.

[60] Manavathi B., Lo D., Bugide S., Dey O., Imren S., Weiss M.J. (2012). Functional regulation of pre-B-cell leukemia homeobox interacting protein 1 (PBXIP1/HPIP) in erythroid differentiation. J. Biol. Chem..

[61] Barbieri M.A., Li G., Mayorga L.S., Stahl P.D. (1996). Characterization of Rab5:Q79L-stimulated endosome fusion. Arch. Biochem. Biophys..

[62] Webb D.J., Donais K., Whitmore L.A., Thomas S.M., Turner C.E., Parsons J.T. (2004). FAK-Src signalling through paxillin, ERK and MLCK regulates adhesion disassembly. Nat. Cell Biol..

[63] Alanko J., Mai A., Jacquemet G., Schauer K., Kaukonen R., Saari M. (2015). Integrin endosomal signalling suppresses anoikis. Nat. Cell Biol..

[64] Kuo J.C., Han X., Yates J.R., Waterman C.M. (2012). Isolation of focal adhesion proteins for biochemical and proteomic analysis. Methods Mol. Biol..

[65] Roberts M., Barry S., Woods A., van der Sluijs P., Norman J. (2001). PDGF-regulated rab4-dependent recycling of alphavbeta3 integrin from early endosomes is necessary for cell adhesion and spreading. Curr. Biol..

[66] Roy A., Kucukural A., Zhang Y. (2010). I-TASSER: a unified platform for automated protein structure and function prediction. Nat. Protoc..

[67] Yang J., Yan R., Roy A., Xu D., Poisson J., Zhang Y. (2015). The I-TASSER suite: protein structure and function prediction. Nat. Methods.

[68] Zhang Y. (2008). I-TASSER server for protein 3D structure prediction. BMC Bioinformatics.

[69] Waterhouse A., Bertoni M., Bienert S., Studer G., Tauriello G., Gumienny R. (2018). SWISS-MODEL: homology modelling of protein structures and complexes. Nucleic Acids Res..

