# Supplementary material for: HPIP and RUFY3 are noncanonical guanine nucleotide exchange factors of Rab5 to regulate endocytosis-coupled focal adhesion turnover
Source: J Biol Chem. 2023 Oct 4;299(11):105311. doi: 10.1016/j.jbc.2023.105311 (PMC10641178; doi:10.1016/j.jbc.2023.105311)
Supplement: Supporting Figures S1–S4 [file mmc1.docx]

**
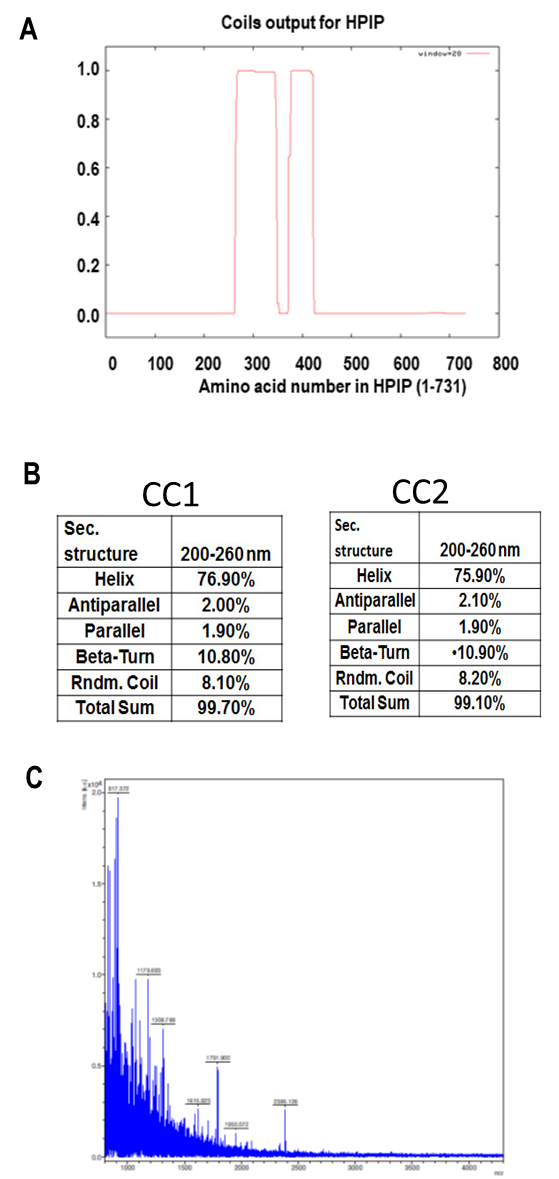
**

**Suppl. Figure S1.** (A) Prediction of coils in HPIP by COILS tool (Expasy). (B) Secondary structure analysis report by CDNN2.1 software on CC1 and CC2 domains. (C) MALDI TOF/TOF spectrum. Peptide Mass Fingerprints (PMF) of RUFY3.


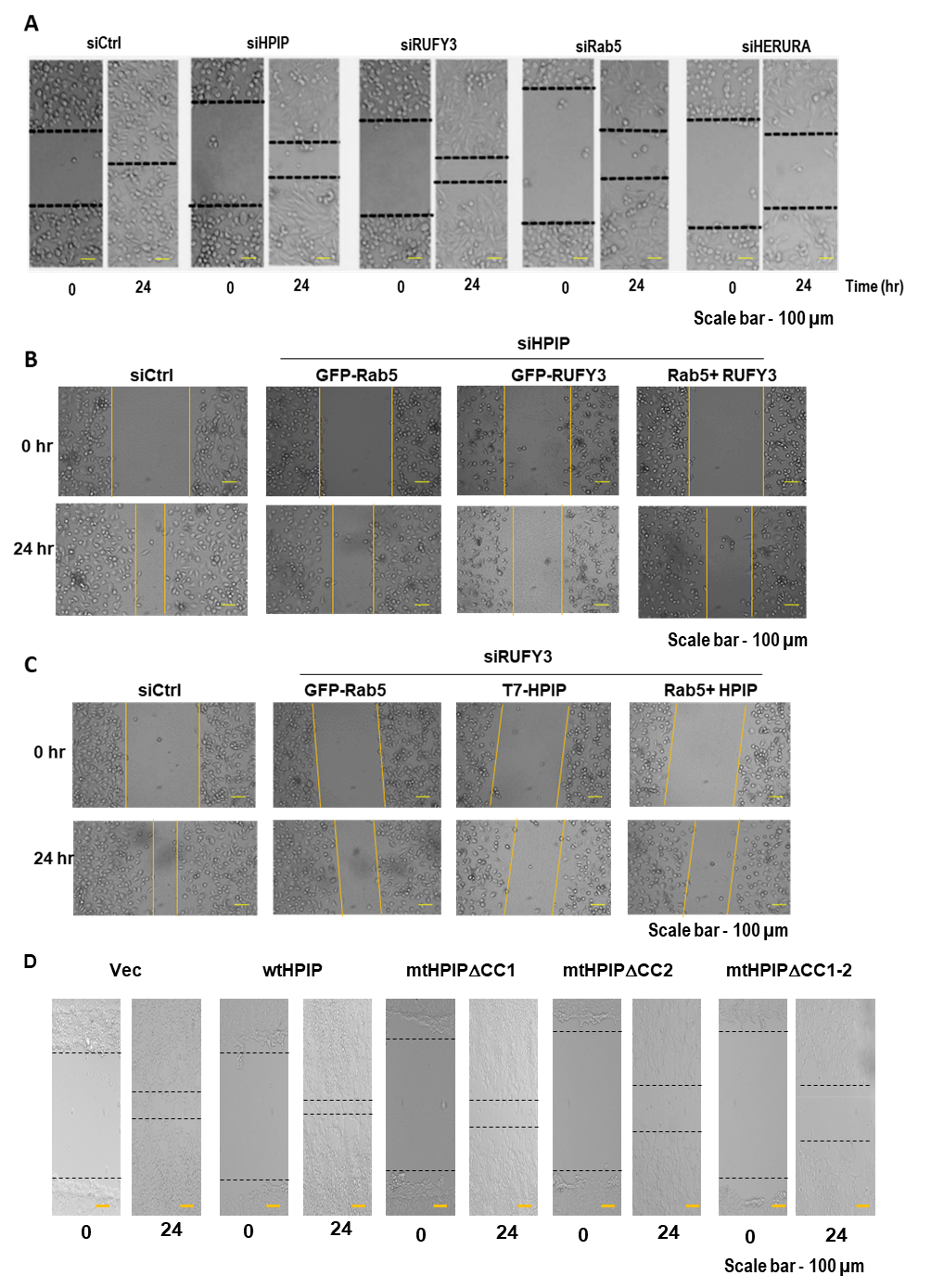


**Suppl. Figure S2.** A. Representative bright field microscopy images (10 x magnifications) of MDA-MB231 cells expressing scrambled control siRNA, siHPIP, siRUFY3, or siRab5 alone or combined depletion of RUFY3, HPIP, and Rab5 (siHERURA) after creating wound and at indicated time points. B. Representative bright field microscopy images (10 x magnifications) of MDA-MB231 cells expressing scrambled control siRNA or siHPIP and GFP-RUFY3 or GFP-Rab5 alone or combined ectopic expression of GFP-RUFY3 and GFP-Rab5 after creating wound and at indicated time points. C. Representative bright field microscopy images (10 x magnifications) of MDA-MB231 cells expressing scrambled control siRNA or siRUFY3 and T7-HPIP or GFP-Rab5 alone or combined ectopic expression of T7-HPIP and GFP-Rab5 after creating wound and at indicated time points. D. Representative bright field microscopy images (10 x magnifications) of MDA-MB231 cells expressing vector control (vec), wtHPIP, mtHPIPΔCC1, mtHPIPΔCC2 or mtHPIPΔCC1-2 after creating wound and at indicated time points. Scale bar, 100 μm


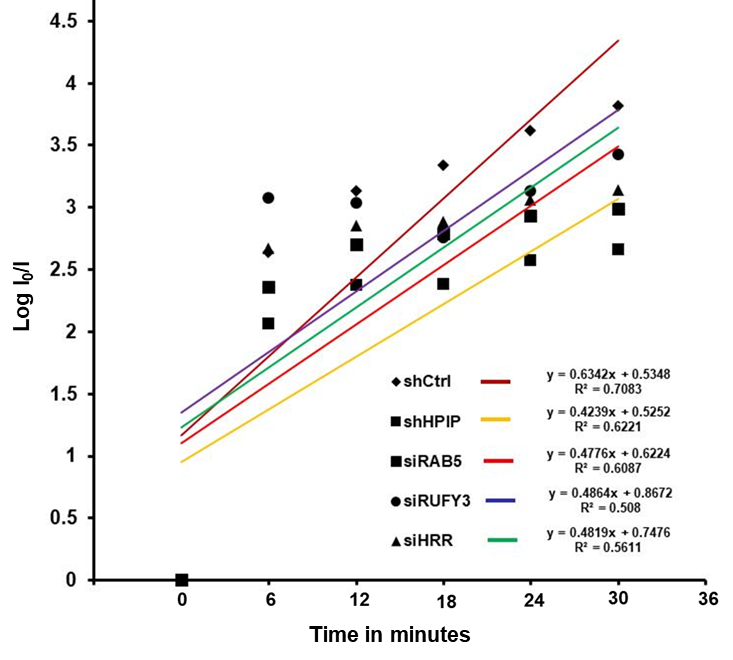


**Suppl. Figure. S3:** Quantification of focal adhesion disassembly rates. Time-lapse sequences in MDA-MB231 cells from live cell fluorescence imaging of DsRed-Paxillin.

A

B


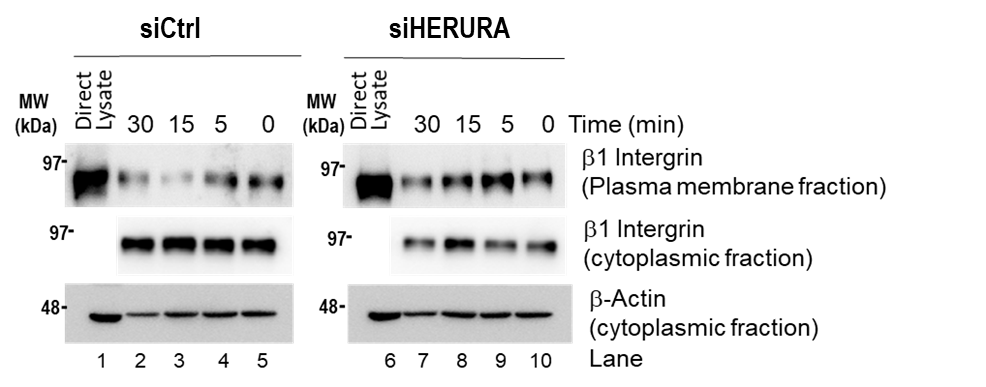


**Suppl. Figure. S4:** HPIP, RUFY3 and Rab5 (HERURA) silencing on β1-integrin internalization. Cellular fractionation analysis demonstrating the levels of β1-intergrin in plasma membrane and cytoplasm in MDA-MB231 cells transfected with control siRNA (siCtrl) or siHPIP, siRUFY3 and siRab5 (siHERURA) at indicated time points. β-Actin was used as cytoplasmic marker. Lane 1 and 6 are direct lysates, whereas 2-5 and 6-9 are respective cellular fractions, such as Plasma membrane or cytoplasm.
