# Supplementary material for: HPIP and RUFY3 are noncanonical guanine nucleotide exchange factors of Rab5 to regulate endocytosis-coupled focal adhesion turnover
Source: J Biol Chem. 2023 Oct 4;299(11):105311. doi: 10.1016/j.jbc.2023.105311 (PMC10641178; doi:10.1016/j.jbc.2023.105311)
Supplement: Supporting Tables S1 and S2 [file mmc2.docx]

| Name | Sequence 5'-3' | Application |
| --- | --- | --- |
| HPIP-CC1 FP | GACATCGGATCCCTTCTGCTGGACAAGCT | subcloning of CC1 into pGEX4T1 vector |
| HPIP-CC1 RP | CTAGCTCGAGTCAGCCCCGGACACAGTCGG |  |
| HPIP-CC2 FP | GACATCGGATCCGAACTCAGCTTCCTGA A | subcloning of CC2 into pGEX4T1 vector |
| HPIP-CC2 RP | CTAGCTCGAGTCACTGCAAGCTCCTCTCCA |  |
| HPIP∆CC1 left fragment FP | GACAGGATCCTGATGGCCTCCTGCCCA | subcloning of HPIP∆CC1 into pcDNA 3.1 vector |
| HPIP∆CC1 left fragment RP | CTAGGATATCACCCATGTTTTGCAG |  |
| HPIP∆CC1 right fragment FP | 5’-GACAGATATCCCCAGATGGGGTGTG | subcloning of HPIP∆CC1 into pcDNA 3.1 vector |
| HPIP∆CC1 right fragment RP | CTAGCTCGAGTGCCCCGGTGGTGGTG |  |
| HPIP∆CC2 left fragment FP | GACAGGATCCTGATGGCCTCCTGCCCA | subcloning of HPIP∆CC2 into pcDNA 3.1 vector |
| HPIP∆CC2 left fragment RP | CTAGGATATCGAG TGCCCCGGTGGTGGTG |  |
| HPIP∆CC2 right fragment FP | GACAGATATCCCAGCCGCGGGGACC | subcloning of HPIP∆CC2 into pcDNA 3.1 vector |
| HPIP∆CC2 right fragment RP | CTAGCTCGAGTGCCCCGGTGGTGGTG |  |
| RUFY3 FP | TAGCCCTCGAGCCACCATGTCTGCCCTGACGCCT | subcloning of RUFY3 into pET28a vector |
| RUFY3 RP | GATCGGGATCCTAATGATGTTTTGGGATCAG |  |
| RUFY3 FP | TAGCCCTCGAGCCACCATGTCTGCCCTGACGCCT | subcloning of RUFY3 into pDsRed N1 vector |
| RUFY3 RP | GATCGGGATCCTAATGATGTTTTGGGATCAG |  |
| Rab5 FP | GCAAAGAATTCATGGCTAGTCGAGGCGCAACA | subcloning of Rab5 into pET28a vector |
| Rab5 RP | GACCCCTCGAGCTAGTTACT ACAACACTGATTCCT |  |
| Rab5-Q79L FP | GATACAGCTGGTCTAGAACGATACCAT | mutation of Q into L in Rab5 |
| Rab5-Q79L RP | ATGGTATCGTTCTAGACCAGCTGTATC |  |

**Table S1**

List of primers used in this study

**Table S2**

List of antibodies used in the study

| **Name of Antibody** | **Company Name** | **Catalog** |
| --- | --- | --- |
| HPIP | Sigma Aldrich | HPA006949 |
| RUFY3 | SCBT | SC-102090 |
| Rab5 | Cell signalling Technology | 2143S |
| GFP | Sigma Aldrich | G1544 |
| FLAG | Cell signalling Technology | 14793 |
| T7 tag | Novagen | 69522 |
| GST | SCBT | SC-138 |
| His tag | SCBT | SC-8036 |
| Talin | SCBT | SC-7534 |
| Paxillin | SCBT | SC-365379 |
| pFAK(Tyr 397) | SCBT | SC-11765-R |
| FAK | Cell signalling Technology | 3285S |
| β1- Integrin | SCBT | SC-13590 |
| β-Tubulin | Sigma Aldrich | **SAB4200715** |
| β- Actin | Cell signalling Technology | 4970S |
| GAPDH | Cell signalling Technology | 5174S |
| Histone H3 | Cell signalling Technology | 4499S |
